# Enhancement of the antigen-specific cytotoxic T lymphocyte-inducing ability in the PMDC11 leukemic plasmacytoid dendritic cell line via lentiviral vector-mediated transduction of the caTLR4 gene

**DOI:** 10.3892/mmr.2015.3685

**Published:** 2015-04-24

**Authors:** MINAMI IWABUCHI, MIWAKO NARITA, TAKAYOSHI UCHIYAMA, SHUNPEI IWAYA, ERI OIWA, YOSHINORI NISHIZAWA, SHIGEO HASHIMOTO, AUDE BONEHILL, NORIYUKI KASAHARA, JUN TAKIZAWA, MASUHIRO TAKAHASHI

**Affiliations:** 1 Laboratory of Hematology and Oncology, Graduate School of Health Sciences, Niigata University, Niigata 951-8518, Japan; 2 Division of Hematology, Nagaoka Red Cross Hospital, Nagaoka, Niigata 951-8518, Japan; 3 Laboratory of Cellular and Molecular Therapy, Department of Physiology and Immunology, Medical School of the Free University Brussels (VUB), Laarbeeklaan 103, 1090 Brussels, Belgium; 4 CURE Vector Core & JCCC Vector Shared Resource Facility, University of California, Los Angeles, CA 90095, USA; 5 Division of Hematology, Endocrinology and Metabolism, Department of Internal Medicine, Faculty of Medicine, Niigata University, Niigata 951-8510, Japan

**Keywords:** leukemic plasmacytoid dendritic cell line, PMDC11, caTLR4, gene transduction, lentiviral vector, antigen-specific cytotoxic T lymphocytes, adoptive cellular immunotherapy

## Abstract

The aim of the present study was to enhance the efficiency of leukemia immunotherapy by increasing the antigen-specific cytotoxic T lymphocyte-inducing ability of leukemia cells. The leukemic plasmacytoid dendritic cell line PMDC05 containing the HLA-A02/24 antigen, which was previously established in our laboratory (Laboratory of Hematology and Oncology, Graduate School of Health Sciences, Niigata University, Niigata, Japan), was used in the present study. It exhibited higher expression levels of CD80 following transduction with lentiviruses encoding the CD80 gene. This CD80-expressing PMDC05 was named PMDC11. In order to establish a more potent antigen-presenting cell for cellular immunotherapy of tumors or severe infections, PMDC11 cells were transduced with a constitutively active (ca) toll-like receptor 4 (TLR4) gene using the Tet-On system (caTLR4-PMDC11). CD8^+^ T cells from healthy donors with HLA-A02 were co-cultured with mutant WT1 peptide-pulsed PMDC11, lipopolysaccharide (LPS)-stimulated PMDC11 or caTLR4-PMDC11 cells. Interleukin (IL)-2 (50 IU/ml) and IL-7 (10 ng/ml) were added on day three of culture. Priming with mutant WT1 peptide-pulsed PMDC11, LPS-stimulated PMDC11 or caTLR4-PMDC11 cells was conducted once per week and two thirds of the IL-2/IL-7 containing medium was replenished every 3–4 days. Immediately prior to the priming with these various PMDC11 cells, the cultured cells were analyzed for the secretion of interferon (IFN)-γ in addition to the percentage and number of CD8^+^/WT1 tetramer^+^ T cells using flow cytometry. caTLR4-PMDC11 cells were observed to possess greater antigen-presenting abilities compared with those of PMDC11 or LPS-stimulated PMDC11 cells in a mixed leukocyte culture. CD8 T cells positive for the WT1 tetramer were generated following 3–4 weeks of culture and CD8^+^/WT1 tetramer^+^ T cells were markedly increased in caTLR4-PMDC11-primed CD8^+^ T cell culture compared with PMDC11 or LPS-stimulated PMDC11-primed CD8^+^ T cell culture. These CD8^+^ T cells co-cultured with caTLR4-PMDC11 cells were demonstrated to secrete IFN-γ and to be cytotoxic to WT1-expressing target cells. These data suggested that the antigen-specific cytotoxic T lymphocyte (CTL)-inducing ability of PMDC11 was potentiated via transduction of the caTLR4 gene. The present study also suggested that caTLR4-PMDC11 cells may be applied as potent antigen-presenting cells for generating antigen-specific CTLs in adoptive cellular immunotherapy against tumors and severe viral infections.

## Introduction

Dendritic cells (DCs) are potent antigen-presenting cells (APCs), which can prime naïve T cells to differentiate into antigen-specific cytotoxic T lymphocytes (CTLs), which are involved in the immune reaction (1). By utilizing this function of DCs, DC-based cellular immunotherapy has been developed for use in anti-tumor therapy and has been approved for use in prostate cancer by the Food and Drug Administration (Silver Spring, MD, USA) (2). In addition to therapy involving direct administration of DCs to patients, adoptive specific cellular immunotherapy has been developed. This involves the infusion of antigen-specific CTLs generated by priming lymphocytes with antigen-presenting DCs *in vitro* into patients, which has been demonstrated to be a promising novel therapeutic treatment strategy in cancer (3). Although previous studies have supported this treatment option, it is technically difficult and labor-intensive to expand what is currently known about antigen-specific CTLs *in vitro*. Adoptive immunotherapy using antigen-specific CTLs may be more practical for anti-cancer treatment if the potent APCs were available at any time.

In a previous study by our group, a leukemic plasmacytoid dendritic cell (pDC) line, PMDC05, was established from pDC leukemia cells (4). The PMDC05 cells were antigen-presenting, which was shown to be enhanced by stimulation with lipopolysaccharides (LPS) (4). Previously, PMDC05 cells were demonstrated to undergo a morphological, surface-phenotypical and functional transformation from pDC to a myeloid DC (mDC) lineage following the stimulation with interleukin (IL)-3 or LPS (5). Furthermore, PMDC05 cells have been indicated to possess a potent ability to generate tumor antigen-specific CTLs from normal peripheral blood (PB) CD8^+^ T cells *in vitro* (6). Yamahira *et al* (7) identified that an antigen-presenting ability equivalent to that of maximally activated PMDC05 could be invoked in PMDC05 cells via lentiviral vector-mediated transduction of CD80 genes. CD80 gene-transduced PMDC05 cells were termed PMDC11 in the present study, and their antigen-presenting ability was potentiated by stimulation with LPS. The present study aimed to establish efficient and potent APCs by transducing the constitutively active (ca) toll-like receptor 4 (TLR4) gene into PMDC11 using the Tet-On system with a lentiviral vector.

## Materials and methods

### Cell culture

PMDC05 cells (4) and PMDC11 cells (7) were established in our laboratory (Laboratory of Hematology and Oncology, Graduate School of Health Sciences, Niigata University, Niigata, Japan). The cells were cultured in Iscove’s modified Dulbecco’s medium (IMDM; Invitrogen Life Technologies, Carlsbad, CA, USA) with 10% fetal bovine serum (FBS; Nichirei Biosciences, Inc., Tokyo, Japan). For establishing the PMDC05 cells, and using both of the cell lines, written informed consent was obtained from the patient’s spouse. The present study was approved by the ethics committee of the Faculty of Medicine, Niigata University (March 25, 2011).

### Lentivirus production and titer determination

Virus production was conducted using transient co-transfection into 293T cells (American Type Culture Collection, Manassas, VA, USA) according to the method described previously (8). The lentiviruses were concentrated by ultracentrifugation at 82,700 × g for 120 min at 4°C, typically resulting in titers of >10^8^ transduction units/ml. The lentiviral titer was determined via the assessment of the viral p24 antigen concentration using an ELISA (Cell Biolabs, Inc., San Diego, CA, USA), and is hereafter expressed as *µ*g of p24 equivalent units/ml. A measure of 1 *µ*g p24 equivalent per ml corresponds to ~1–5×10^7^ transduction units/ml using 293T cells as a reference line. p24 concentrations of pLVX-Tet-ON Advanced and pLVX-Tight-TLR4-IRES-GFP-PGK-Puro lentiviruses (Clontech Laboratories, Inc., Mountain View, CA, USA) used in the present study were 19.1 and 11.3 *µ*g/ml, respectively. The lentiviruses were prepared by Dr Noriyuki Kasahara (CURE Vector Core & JCCC Vector Shared Resource Facility, University of California, Los Angeles, CA, USA).

### Lentiviral vector transduction

Transduction of pLVX-Tet-ON Advanced vector into the PMDC11 cells was performed as previously described (9). A total of 5×10^5^ PMDC11 cells were suspended in a 15-ml tube containing 200 *µ*l lentiviral vector (19.1 *µ*g/ml p24 core protein by ELISA) and protamine sulfate (Sigma-Aldrich, St. Louis, MO, USA) at a final concentration of 5 *µ*g/ml. Cells were centrifuged at 800 × g for 30 min and suspended in 800 *µ*l 10% FBS-containing IMDM, and were subsequently transferred to a 12-well plate. The 12-well plate containing the cells was centrifgued at 800 × g for 30 min (PlateSpinII; Kubota, Tokyo, Japan), in order to enhance the transduction efficiency, and the cells were then incubated at 37°C overnight in the presence of 5% CO_2_. Cells were then carefully collected in 15-ml tubes and diluted with fresh media and centrifuged at 200 × g for 5 min. The supernatant was removed and 2 ml fresh medium was added to the tube. Cells were then transferred into a 35-mm culture dish and cultured for 24 h. PMDC11 cells transduced with pLVX-Tet-ON Advanced vector were selected by adding 400 mg/ml G418 (Sigma-Aldrich) to the culture dish on the next day, followed by further culturing for 7–12 days. G418-selected PMDC11 cells were transduced with pLVX-Tight-TLR4-IRES-GFP-PGK-Puro lentiviral vector using an almost identical method, with the exception of using two selection reagents [1 *µ*g/ml puromycin (Sigma-Aldrich) and G418]. The concentrations of G418 and puromycin used in the present study were confirmed to be appropriate for PMDC11 cells by a titration assay (10). The selected PMDC11 cells were termed caTLR4-PMDC11.

### Evaluation of Tet-On system

The operation of the lentiviral Tet-On system with pLVX-Tet-ON Advanced vector and pLVX-Tight-TLR4-IRES-GFP-PGK-Puro vector was evaluated by culturing caTLR4-PMDC11 cells in G418/puromycin-containing medium with various concentrations of doxycycline for 6 h to 3 days and analyzing the expression of green fluorescent protein (GFP) on doxycycline-exposed caTLR4-PMDC11 cells using a FACSCalibur flow cytometer (BD Biosciences, San Jose, CA, USA).

### Stimulation of PMDC11 and caTLR4-PMDC11

PMDC11 cells (1.0×10^6^ cells/ml) were stimulated with 0.1 *µ*g/ml LPS for 24 h (PMDC11 + LPS), whereas caTLR4-PMDC11 cells (1.0×10^6^ cells/ml) were activated with 1 *µ*g/ml doxycycline for 24 h (caTLR4-PMDC11^*^Dox) prior to further analysis.

### Phenotype analysis

PMDC11 cells and caTLR-PMDC11 cells were stained with the following phycoerythrin (PE)-conjugated monoclonal antibodies: mouse CD1a (cat. no. 1590; Immunotech, Marseille, France), rat CD40 (cat. no. 732138; Immunotech), mouse CD80 (cat. no. 560925; BD Biosciences), mouse CD83 (cat. no. 305307; BioLegend, San Diego, CA, USA), mouse CD86 (cat. no. 305405; BioLegend) and mouse HLA-DR (cat. no. 307605; BioLegend). Stained cells were assessed using the FACSCalibur flow cytometer and data were analyzed using CellQuestPro software (BD Biosciences).

### Allogeneic mixed leukocyte culture (MLC)

MLC was performed as described previously (4). Briefly, PMDC11 cells or caTLR4-PMDC11 cells were irradiated with 30 Gy of ^137^Cs generated gamma irradiation (PS-3000SB Cs-137; Pony Industry Co., Ltd., Osaka, Japan) immediately prior to MLC. A total of 1×10^5^ allogeneic peripheral blood mononuclear cells (PB-MNCs) were co-cultured in a 96-well flat-bottom microtiter plate (BD Biosciences) with graded numbers (0, 1,250, 2,500 and 5,000) of irradiated PMDC11 cells or caTLR4-PMDC11 cells. The co-cultured cells were pulsed with 0.5 *µ*Ci (18.5 KBq)/well of [methyl-^3^H]-thymidine (Perkin-Elmer, Inc., Waltham, MA, USA) on day five of culture and harvested with a cell harvester (Labo Mash; Futaba Medical, Inc., Tokyo, Japan) following overnight culture. Cellular proliferation was measured by ^3^H-thymidine incorporation with a liquid scintillation counter (LSC-5100; Hitachi Aloka Medical Ltd., Tokyo, Japan). The experiments were conducted in triplicate.

### Interferon (IFN)-γ assay using cytometric bead array (CBA)

IFN-γ in the MLC supernatant was quantified using the CBA Human IFN-γ Flex Set Assay (BD Biosciences) according to the manufacturer’s instructions. Briefly, the provided standard IFN-γ (BD Biosciences) or culture supernatant was added to IFN-γ Capture Beads and the mixture was incubated for 1 h. PE-conjugated anti-human IFN-γ antibody (IFN-γ PE Detection Reagent) was added to the mixture, which was then incubated for 2 h. The mixture was washed and centrifuged at 300 × g for 10 min at room temperature. The centrifuged pellet was then resuspended in 300 ml wash buffer. The FACSCalibur flow cytometer was set up with BD FACSComp version 5.1 software and BD Calibrite beads (BD Biosciences). IFN-γ concentrations were indicated by their fluorescent intensities (Fl-2) and were quantified using the standard reference curve provided for IFN-γ.

### CTL induction using PMDC11 or caTLR4-PMDC11

CD8^+^ T cells were separated from PB-MNCs of a normal person with HLA-A*24:02 using the fluorescein isothiocyanate (FITC)-conjugated CD8 monoclonal antibody and anti-FITC microbeads (Miltenyi Biotec GmbH, Bergisch Gladbach, Germany) according to the manufacturer’s instructions. PMDC11 cells and caTLR4-PMDC11 cells were incubated with 10 mg/ml mutant (m) 9mer WT1 peptides with antigenicity in the HLA-A*24:02^+^ individual (CYTWNQMNL; NeoMPS, Inc., San Diego, CA, USA) for 24 h. caTLR4-PMDC11 cells were exposed to 1 mg/ml doxycycline during this incubation period for operating Tet-On system. CD8^+^ T cells (5×10^5^/well) were co-cultured with 30 Gy-irradiated PMDC11 cells or caTLR4-PMDC11 cells at a cell ratio of 2:1 in a six-well plate containing 2.5 ml 5% autologous serum-containing RPMI 1640 medium (Invitrogen Life Technologies). IL-2 (Shionogi & Co., Ltd., Osaka, Japan) and IL-7 (R&D Systems, Inc., Minneapolis, MN, USA) were added to the co-culture on day three at a final concentration of 50 U/ml and 10 ng/ml, respectively. A proportion of two thirds of the medium containing IL-2 and IL-7 was replenished every 2–3 days throughout the culture period. The co-culture was stimulated repeatedly every week with the same mWT1 peptide-pulsed PMDC11 cells or caTLR4-PMDC11 cells. mWT1 tetramer analysis of the co-cultured lymphocytes was performed every week immediately prior to the addition of PMDC cells (11).

### Cytotoxicity assay of co-cultured CD8^+^ T cells

A cytotoxicity assay was conducted using flow cytometry with co-cultured CD8^+^ T cells as effector cells and T2A24 cells [transporter associated with antigen processing-deficient, HLA-A24-expressing human T, B-hybridoma cells] as the target cells. The T2A24 cells were donated by Dr Yoshiki Akatsuka (Department of Hematology, Fujita Health University, Toyoake, Aichi, Japan). Lentivirally GFP gene-transduced T2A24 cells, which were pulsed with mWT1 peptides for 24 h, were cultured with effector cells in RPMI 1640 without phenol red for 4 h at 37°C in a fully humidified 5% CO_2_ atmosphere. Co-cultured cells (consisting of effector cells and target cells) were stained with 7-amino-actinomycin D (7AAD; Sigma-Aldrich) immediately prior to flow cytometric analysis. Cells were acquired for 120 sec in all the samples and viable target cells (GFP^+^/7AAD^−^) were gated in an FL-1 (GFP)/FL-3 (7AAD) dot plot. The percentage cytotoxicity of the assay was calculated with the following formula: % Cytotoxicity=[(absolute number of viable target cells in the tube containing target cells only - absolute number of viable target cells in the sample tube)/absolute number of viable target cells in the tube containing target cells only] ×100.

### Statistical analysis

The statistical significance of differences between values was evaluated via one- and two-way analysis of variance, with GraphPad Prism version 5 software (GraphPad Software, Inc., La Jolla, CA, USA). P<0.05 was considered to indicate a statistically significant difference.

## Results

### Antigen presentation-associated molecules of caTLR4-PMDC11 cells

With regard to the establishment of the conditions for driving the Tet-On system, caTLR4 was observed to be highly expressed on caTLR4-PMDC11 following 24-h incubation with 1 µg/ml doxycycline, in the presence of which caTLR4-PMDC11 cells were viable. Therefore, caTLR4 expression was induced with 1 *µ*g/ml of doxycycline for 24 h in the subsequent experiments.

**Figure 1:**
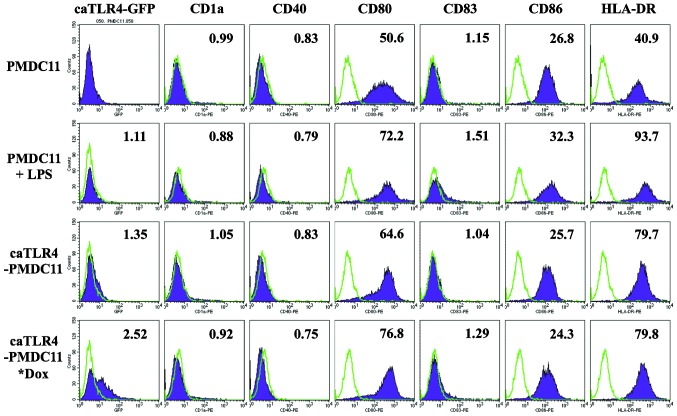
Expression of antigen presentation-associated molecules in PMDC11 and caTLR4-PMDC11 cells. PMDC11, LPS-stimulated PMD11, caTLR4-PMDC11 and doxycycline-exposed caTLR4-PMD11 cells were analyzed for the expression of antigen presentation-associated molecules by flow cytometry. Histograms representative of three experiments are presented. PMDC11 and caTLR4-PMDC11 cells were treated with LPS (0.1 *µ*g/ml) or doxycycline (1 *µ*g/ml) for 24 h, respectively. Filled purple histograms represent staining with specific antibodies and open green histograms represent isotype-matched controls. Values in the graphs indicate mean fluorescence intensities. LPS, lipopolysaccharide; Dox, doxycycline; GFP, green fluorescent protein.

The expression of antigen presentation-associated molecules, including CD80, CD83, CD86 and HLA-DR, on PMDC11 cells was demonstrated to be enhanced by the stimulation with LPS for 24 h. caTLR4-PMDC11 cells without doxycycline exposure exhibited a marginal increase in CD80 and HLA-DR expression, which was suggested to be due to a spontaneous and partial expression of caTLR4 in addition to GFP when the Tet-On system was inactive. By driving the Tet-On system with doxycycline in caTLR4-PMDC11, which was confirmed by the expression of GFP, the expression of CD80, CD83 and HLA-DR was enhanced to the level of LPS-stimulated PMDC11 cells (Fig. 1).

### Antigen-presenting ability of caTLR-PMDC11

**Figure 2:**
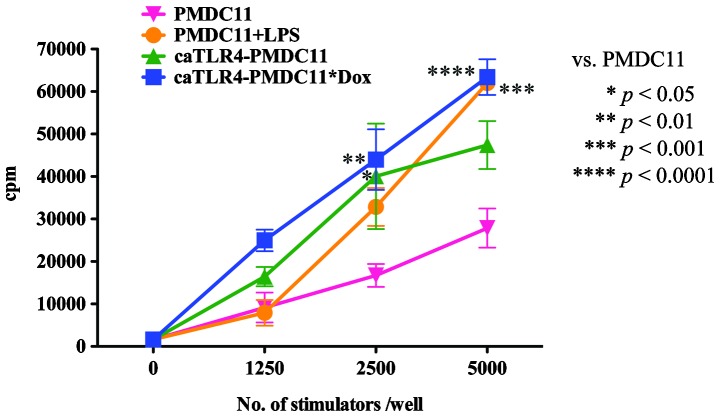
Antigen-presenting ability of PMDC11 and caTLR-PMDC11 cells. Antigen-presenting abilities of these stimulator cells were assayed by allogeneic mixed leukocyte culture using ^3^H-thymidine incorporation. Graded numbers of PMDC11, LPS-stimulated PMD11, caTLR4-PMDC11 or doxycycline-exposed caTLR4-PMD11 cells were used as stimulator cells and normal peripheral blood mononuclear cells (1×10^5^/well) were the responder cells. Statistical differences between PMDC11 cells and other stimulator cells were evaluated by two-way analysis of variance. The values are expressed as the mean ± standard error of the mean. The two experiments not presented had similar results. cpm, count per minute; LPS, lipopolysaccharide; Dox, doxycycline.

The antigen-presenting ability of caTLR4-PMDC11 cells with or without doxycycline exposure was compared with that of PMDC11 cells with or without LPS stimulation by performing MLC with ^3^H-thymidine incorporation. The antigen-presenting ability of PMDC11 cells was identified to be enhanced by stimulation with LPS. By transduction with the caTLR4 gene, the antigen-presenting ability of PMDC11 cells was observed to increase (caTLR4-PMDC11 without doxycycline exposure). In addition, following exposure to doxycycline, the antigen-presenting ability of caTLR4-PMDC11 was increased. caTLR4-PMDC11 cells possessed greater than or equal antigen-presenting ability compared with that of the LPS-stimulated PMDC11 cells (Fig. 2). This enhanced antigen-presenting ability of caTLR4-PMDC11 cells without doxycycline exposure compared with that of PMDC11 cells was hypothesized to be due to spontaneous and partial expression of caTLR4 in the state of Tet-On system inactivity.

### IFN-γ production of caTLR-PMDC11

**Figure 3:**
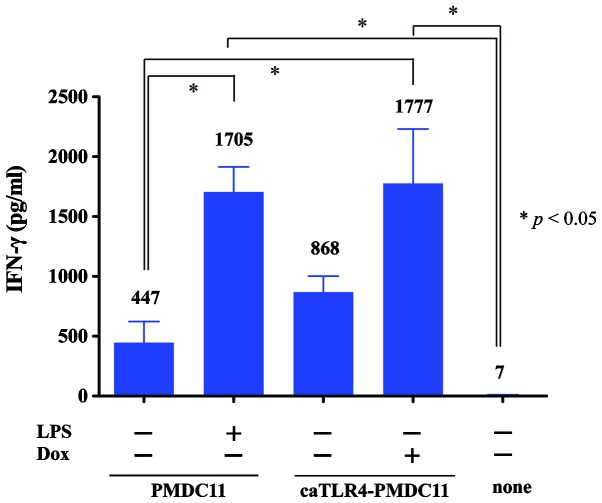
IFN-γ production of PB-MNCs stimulated with PMDC11 or caTLR-PMDC11 cells. The IFN-γ concentration of MLC supernatant was assayed using the cytometric bead array human IFN-γ Flex Set Assay. A total of 1×10^4^ cells/well of PMDC11, LPS-stimulated PMD11, caTLR4-PMDC11 or doxycycline-exposed caTLR4-PMD11 cells were used as stimulator cells and 1×10^5^ cells/well of normal PB-MNCs were used as responder cells in MLC over 5 days. The bar graph shows the mean concentration of IFN-γ. Statistical differences between groups were evaluated using one-way analysis of variance. The values are expressed as the mean ± standard error of the mean. IFN, interferon; PB-MNCs, peripheral blood mononuclear cells; MLC, mixed leukocyte culture; LPS, lipopolysaccharide; Dox, doxycycline.

IFN-γ production of lymphocytes stimulated by caTLR4-PMDC11 cells with or without doxycycline exposure was compared with that produced by PMDC11 cells with or without LPS stimulation by using the CBA Human IFN-γ Flex Set assay (Fig. 3). IFN-γ production of PMDC11-stimulated lymphocytes was observed to be enhanced by the culture of PMDC11 cells with LPS. By transduction with the caTLR4 gene, the lymphocyte-stimulating ability of PMDC11 cells leading to the production of IFN-γ was increased (caTLR4-PMDC11 without doxycycline exposure). In addition, in the presence of doxycycline, the lymphocyte-stimulating ability of caTLR4-PMDC11 cells leading to the production of IFN-γ was demonstrated to increase. Doxycycline-treated caTLR4-PMDC11 cells possessed equivalent levels of potency in mediating the production of IFN-γ by lymphocytes compared with those of LPS-stimulated PMDC11 cells (Fig. 3). The enhanced lymphocyte-stimulating ability of caTLR4-PMDC11 without doxycycline exposure leading to the production of IFN-γ compared with that of PMDC11 cells was suggested to be due to spontaneous and partial expression of caTLR4 in the state of Tet-On system inactivity.

### mWT1-specific CTL induction by caTLR4-PMDC11 cells

**Figure 4:**
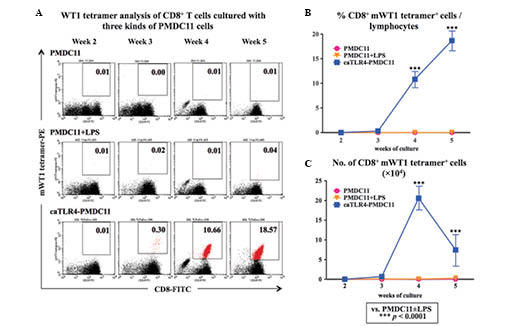
mWT1-specific cytotoxic T-lymphocyte induction by caTLR4-PMDC11 cells. Normal PB-CD8^+^ T cells were cultured with mWT1 peptide-pulsed PMDC11, LPS-stimulated PMDC11 or doxycycline-exposed caTLR4-PMDC11 cells for five weeks. The co-cultured cells were double stained with the FITC-conjugated CD8 antibody and PE-conjugated mWT1 tetramer. (A) mWT1 tetramer analysis of CD8^+^ T cells cultured with PMDC11, LPS-stimulated PMDC11 or doxycycline-exposed caTLR4-PMDC11 cells. Values in the dot plots represent the percentages of mWT1 tetramer^+^/CD8^+^ T cells (marked in red) among the cells in the lymphocyte gate of forward scatter/side scatter dot plots. Dot plots are representative of three experiments with similar results. (B) Percentages of mWT1 tetramer^+^/CD8^+^ T cells in the lymphocyte gate of the forward scatter/side scatter dot plots. (C) Total number (×10^4^) of CD8^+^ mWT1 tetramer^+^ cells in a well, which was calculated by the formula [(percentage of mWT1 tetramer^+^/CD8^+^ T cells among lymphocytes) × (total number of lymphocytes in culture well)]. LPS, lipopolysaccharide; FITC, fluorescein isothiocyanate; PE, phycoerythrin.

The mWT1-specific CTL-inducing ability of normal PB-CD8^+^ T cells was compared between the PMDC11, LPS-stimulated PMDC11 and doxycycline-treated caTLR4-PMDC11 cells as the stimulator cells. Following three to five weeks of co-culture of CD8^+^ T cells and PMDC11 cells, doxycycline-treated caTLR4-PMDC11 cells generated an increased percentage and number of mWT1-specific CTLs compared with that of PMDC11 or LPS-stimulated PMDC11 cells (Fig. 4).

### Cytotoxicity of mWT1-specific CTLs induced by caTLR4-PMDC11

**Figure 5:**
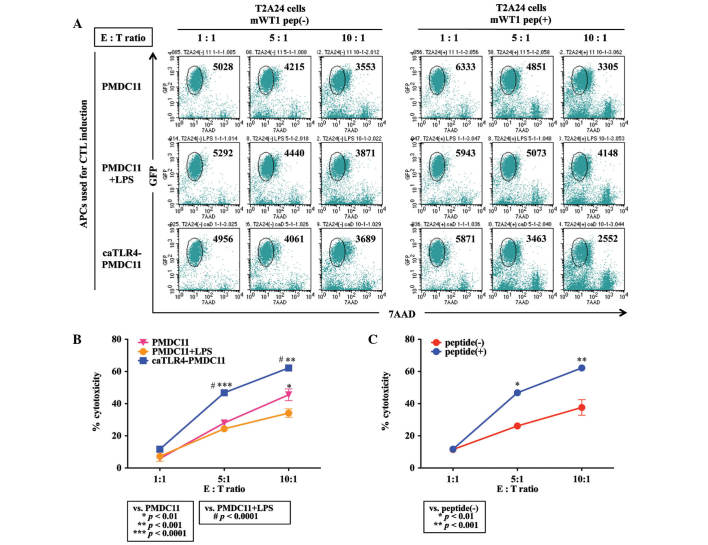
Cytotoxicity of mWT1-specific CTLs induced by caTLR4-PMDC11. mWT1-specific cytotoxicity of CTLs generated from normal CD8^+^ T cells by priming with mWT1 peptide-pulsed PMDC11, LPS-stimulated PMDC11 or doxycycline-exposed caTLR4-PMDC11 cells was evaluated using GFP-transduced T2A24 cells pulsed with or without mWT1 peptide as target cells by flow cytometric analysis. Target cells were cultured with effector cells in RPMI 1640 without phenol red for 4 h at 37°C. Co-cultured cells were stained with 7AAD and subjected to flow cytometric analysis. Cells were acquired for 120 sec and viable target cells (GFP^+^/7AAD^−^) were counted. The cytotoxicity was calculated using the following formula: [(absolute number of viable target cells in the tube containing target cells only - absolute number of viable target cells in the sample tube)/absolute number of viable target cells in the tube containing target cells only] ×100%. (A) Flow cytometry-based cytotoxicity assay of CD8^+^ T cells cultured with three types of PMDC11 cells. Gated cells in the dot plots represent the number of viable target cells; two repetitions of the experiment produced similar results. (B) Cytotoxicity of CD8^+^ T cells cultured with three types of PMDC11 cells against mWT1 peptide-pulsed T2A24 cells. (C) Cytotoxicity of CD8^+^ T cells cultured with caTLR4-PMDC11 cells against T2A24 cells pulsed with or without mWT1 peptide. Statistical differences between the two experimental groups were evaluated by two-way analysis of variance. The values are expressed as the mean ± standard error of the mean. APC, antigen-presenting cell; CTL, cytotoxic T lymphocyte; LPS, lipopolysaccharide; GFP, green fluorescent protein; 7AAD, 7-aminoactinomycin D.

Antigen-specific cytotoxicity of CTLs generated from normal CD8^+^ T cells by priming with mWT1 peptide-pulsed PMDC11 cells, LPS-stimulated PMDC11 cells or doxycycline-exposed caTLR4-PMDC11 cells was evaluated by flow cytometric analysis using mWT1 peptide-pulsed and GFP-transduced T2A24 cells as target cells. CD8^+^ T cells co-cultured with mWT1 peptide-pulsed caTLR4-PMDC11 cells for four weeks demonstrated an increased cytotoxicity against mWT1 peptide-pulsed target cells compared with that of CD8^+^ T cells co-cultured with mWT1 peptide-pulsed PMDC11 or LPS-stimulated PMDC11 cells (Fig. 5A and B). The differences among the PMDC11, LPS-stimulated PMDC11 and doxycycline-exposed caTLR4-PMDC11 cells were suggested to be due to the increased percentage of mWT1-specific CTLs present in the co-cultured cells. Although non-specific cytotoxicity against T2A24 cells without mWT1 peptide loading was observed in CD8^+^ T cells co-cultured with mWT1 peptide-pulsed caTLR4-PMDC11 cells, the percentage cytotoxicity against T2A24 cells loaded with the mWT1 peptide was significantly greater than that of non-loaded target cells at E:T ratios of 5:1 and 10:1 (P<0.01; Fig. 5C).

## Discussion

Freely available APCs with standardized quality are required for the establishment of an efficient adoptive cellular immunotherapy method for use in tumors and severe viral infections. Although autologous monocyte-derived DCs (moDCs) are commonly used as APCs for use in immunotherapy, the preparation of a sufficiently large quantity is complex and the quality of moDCs varies between individuals (12). Artificial APCs using either acellular (13–16) or cell-based systems [e.g., *Drosophila* (17), mouse fibroblast (18,19), human erythro-myeloid (20,21) or breast carcinoma (22) cell lines] have been introduced in place of moDCs. In these cell-based artificial APCs, antigen presentation-associated molecules, including major histocompatibility class I (17–21), CD80 (17–19,21,22), intercellular adhesion molecule 1 (CD54) (17–19,21), lymphocyte function-associated antigen 3 (CD58) (18,19,21) or CD83 (21) require expression via cDNA transfection in order to reach sufficient levels of antigen-presenting ability on these artificial APCs.

Additionally, a DC line with antigen-presenting ability may be used for the induction of anti-tumor or anti-viral CTLs. In order to do this, three cell lines are available, one of which is the PMDC05 cell line, which is derived from pDC leukemia cells. Another is a pDC line, GEN2.2, which has been reported to require a monolayer of irradiated (60 Gy) MS-5 feeder cells for growth (23); however, an additional study has suggested that it may be able to grow without feeder cells (24). The third cell line is CAL-1, which was originally established from a patient with blastic natural killer cell lymphoma. CAL-1 cells are very similar to PMDC05 cells in morphology and surface phenotypes, in addition to producing a small quantity of IFN-α (25). Recently, CAL-1 was used for evaluating the efficacy of anti-nuclear factor (NF)-κB-treatment on blastic plasmacytoid dendritic cell neoplasms, which was associated with aberrant activation of the NF-κB pathway (26). With regard to GEN2.2, Aspord *et al* (27) demonstrated that the GEN2.2 cell line was able to stimulate increased levels of tumor/viral antigen-specific and functional cytotoxic T cell responses *in vitro*. They additionally noted that this cell line resulted in an inhibition of tumor growth in a humanized mouse *in vivo* model. Furthermore, Aspord *et al* (27) indicated that the GEN2.2 vaccine had a greater efficacy than conventional myeloid DC-based vaccines and suggested that an allogeneic pDC line-based vaccine may be a novel therapeutic tool for clinical application in cancer treatment (27). GEN2.2 cells have additionally been observed to secrete Type I and III IFN in response to the hepatitis C virus pathogen-associated molecular patterns (28).

PMDC05 cells, which were established in our laboratory (Laboratory of Hematology and Oncology, Graduate School of Health Sciences, Niigata University), are APCs and are able to produce a small quantities of IFN-α when stimulated with CpG and influenza virus (29). By stimulation with LPS, PMDC05 cells produce IL-12p70 and the antigen-presenting ability is markedly increased (5). PMDC05 cells are able to induce antigen-specific CTLs efficiently from normal PB-CD8^+^ T cells in the co-culture experiments using tumor antigen (mWT1 peptide) or viral antigen (CMVpp65 peptide) (6). Although PMDC05 cells have an antigen-presenting ability without stimulation, minimal expression of CD80 was detected on PMDC05 cells (4). Therefore PMDC05 cells were transduced with the CD80 gene using a lentiviral vector and the CD80-transduced PMDC05 cells were termed PMDC11 cells, which were demonstrated to have a more potent antigen-presenting ability than that of PMDC05 cells (7).

Preliminary investigations demonstrated that the expression of antigen presentation-associated molecules and antigen-presenting ability were enhanced in PMDC11 cells via the activation of TLR4 by LPS. To further potentiate the antigen-presenting ability and antigen-specific CTL-inducing ability, the caTLR4 gene was transduced into PMDC11 cells in order to constitutively activate TLR4 signaling while avoiding the use of LPS. LPS is not preferable in a clinical setting due to its gram-negative bacterial endotoxin-origin.

With regard to caTLR4, Cisco *et al* (30) reported that transfection of caTLR4 RNA into DCs induced DC maturation comparable to that driven by the potent cytokine mixture of TNF-α, IL-6, IL-1β and prostaglandin E2. caTLR4 RNA-transfected DCs secreted IL-12 and TNF-α and maturation marker expression was enhanced in them, resulting in an increase in allostimulatory and antigen-specific T-cell responses (30). Abdel-Wahab *et al* (31) demonstrated that co-transfection into DC with RNA of caTLR4 and tumor antigens induced tumor antigen-specific T-cell responses more efficiently than transfection of tumor antigen RNA in combination with maturation cytokines. Bonehill *et al* (32) reported an increase in T-cell stimulatory capacity of moDCs by co-electroporation with differing combinations of CD40L, CD70 and caTLR4-encoding mRNA.

In order to further potentiate the antigen-presenting and CTL-inducing ability of CD80-expressing PMDC11, a lentiviral vector expressing caTLR4 was transduced into PMDC11 cells. PMDC11 cells, which were transduced with the caTLR4-expressing vector, proliferated transiently for three weeks and then proceeded to apoptosis. Thus, PMDC11 cells were subsequently transduced with a caTLR4 gene regulated by the Tet-On system (caTLR4-PMDC11). Normal CD8^+^ T cells were co-cultured with mWT1 peptide-pulsed PMDC11 or caTLR4-PMDC11 and primed with mWT1 peptide-pulsed PMDC11 or caTLR4-PMDC11 every week. mWT1 tetramer^+^/CD8^+^ T cells were generated subsequent to 2–3 weeks of culture and were observed to be significantly increased in caTLR4-PMDC11-primed CD8^+^ T-cell culture, compared with PMDC11-primed CD8^+^ T-cell culture. Identical results were obtained in experiments using CMVpp65 peptide instead of mWT1 peptide (data not shown).

In conclusion, the results of the present study indicated that the antigen-specific CTL-inducing ability of PMDC11 was enhanced by caTLR4 gene transduction and that caTLR4-PMDC11 cells may be applied as potent APCs in order to generate antigen-specific CTLs for adoptive cellular immunotherapy against tumors and severe viral infections.
